# The Impact of Variant Allele Frequency in EGFR Mutated NSCLC Patients on Targeted Therapy

**DOI:** 10.3389/fonc.2021.644472

**Published:** 2021-03-30

**Authors:** Alex Friedlaender, Petros Tsantoulis, Mathieu Chevallier, Claudio De Vito, Alfredo Addeo

**Affiliations:** ^1^ Oncology Department, University Hospital Geneva, Geneva, Switzerland; ^2^ Pathology Department, University Hospital Geneva, Geneva, Switzerland

**Keywords:** TKI, allele frequency, EGFR, NSCLC, VAF, allelic frequency

## Abstract

EGFR mutations represent the most common currently targetable oncogenic driver in non-small cell lung cancer. There has been tremendous progress in targeting this alteration over the course of the last decade, and third generation tyrosine kinase inhibitors offer previously unseen survival rates among these patients. Nonetheless, a better understanding is still needed, as roughly a third of patients do not respond to targeted therapy and there is an important heterogeneity among responders. Allelic frequency, or the variant EGFR allele frequency, corresponds to the fraction of sequencing reads harboring the mutation. The allelic fraction is influenced by the proportion of tumor cells in the sample, the presence of copy number alterations but also, most importantly, by the proportion of cells within the tumor that carry the mutation. Mutations that occur early in tumor evolution, often called clonal or truncal, have a higher allelic frequency than late, subclonal mutations, and are more often drivers of cancer evolution and attractive therapeutic targets. Most, but not all, EGFR mutations are clonal. Although an exact estimate of clonal proportion is hard to derive computationally, the allelic frequency is readily available to clinicians and could be a useful surrogate. We hypothesized that tumors with low allelic frequency of the EGFR mutation will respond less favorably to targeted treatment.

## Highlights

We present a retrospective analysis of the impact of allelic frequency on survival in patients with EGFR mutant non-small cell lung cancer. We then combine allelic frequency with the presence of co-mutations, a known negative predictive factor for targeted therapy in this setting.

## Introduction

Lung cancer is the most commonly diagnosed malignancy worldwide and the leading cause of cancer-related mortality (1). It comprises NSCLC, accounting for 85% of all diagnoses and SCLC. In the last decade, there has been a dramatic surge in the use of targeted therapy, which consists of identifying tumor driving alterations and using small tyrosine kinase inhibitors to block the oncogenic signals (2).

The most common current therapeutic target in lung adenocarcinoma (ADC) consists of epidermal growth factor receptor (EGFR) mutations (3).

EGFR is a monomeric transmembrane receptor tyrosine kinase controlling major molecular pathways of cellular proliferation (4). Upon activation, EGFR phosphorylates tyrosine residues in the intracellular domain, dimerizing and activating downstream signaling including RAS-RAF-MAPK-MEK, STAT, and PI3K-AKT-mTOR pathways, leading to cellular division and proliferation (4).

Activating EGFR mutations in ADC are most common among non-smokers, and younger, female, Asian lung cancer patients (5). The prevalence of EGFR mutations has a significant ethnic variation. They occur in roughly 15% of Caucasian any-stage ADC patients, according to the TCGA, but 22-62% of East Asians with stage III/IV ADC (6). Among East Asian never smokers, EGFR mutations can be found in approximately 80% of advanced lung ADC patients. Furthermore, among a cohort of metastatic, multi-treated, predominantly Caucasian, ADC cases, EGFR was detected in 27% of patients, suggesting that mutations are more common in advanced disease (7).

There are many subtypes of EGFR mutations in ADC, though exon 19 microdeletions and exon 21 point-substitutions comprise 90% of these (8). These common pathogenic variants are highly responsive to small molecule tyrosine kinase inhibitors (TKIs).

Nearly a decade ago, TKIs became standard first-line therapy for EGFR mutant NSCLC. First-generation (erlotinib, gefitinib) and second-generation (afatinib, dacomitinib) TKIs yielded superior outcomes and lower toxicity compared to chemotherapy doublets (9). The appearance of on-target resistance mechanisms, namely exon 20 T790M mutations, prompted the development of osimertinib, a third-generation EGFR TKI. Using the latter upfront was subsequently proven to be superior to prior generation TKIs, both in in terms of progression free survival (PFS) and overall survival (OS) (10). Yet, not all patients derive a similar benefit from TKIs, regardless of the generation of the therapy.

Today, the gold standard for detecting EGFR alterations is through next generation sequencing (NGS), allowing for the detection of a wide panel of oncogenic drivers, including numerous EGFR variants, as well as quantifying the alterations (11). Tumors with oncogenic drivers, such as EGFR in NSCLC, usually depend on a single activated oncogene (12). It yields a survival advantage in this isolated cell line, explaining the low tumor mutation burden (TMB) commonly associated with these diseases (13). The lack of acquired neoantigens through mutations provides a less inflammatory microenvironment and poor in tumor-infiltrating CD8+ lymphocytes (14). This likely, in part, explains why EGFR mutant NSCLC is less sensitive to immune check-point inhibition, which is not part of the front-line therapeutic algorithm for these patients.

In a previous paper, we established that co-occurring genomic mutations may explain the lack of efficacy among a subset of these patients (15). When performing NGS we also calculate the allelic frequency, or mutant allele frequency, corresponding to the fraction of sequencing reads harboring the mutation. The allelic fraction is influenced by the proportion of tumor cells in the sample, the presence of copy number alterations but also, most importantly, by the proportion of cells within the tumor that carry the mutation. Mutations that occur early in tumor evolution, often called clonal or truncal, have a higher allelic frequency than late, subclonal mutations, and are more often drivers of cancer evolution and attractive therapeutic targets (16). Most, but not all, *EGFR* mutations are clonal (17). Although an exact estimate of clonal proportion is hard to derive computationally, the allelic frequency is readily available to clinicians and could be a useful surrogate. We hypothesized that tumors with low allelic frequency of the *EGFR* mutation will respond less favorably to targeted treatment.

## Methods

We identified all patients treated with front-line TKIs (gefitinib, erlotinib, afatinib, osimertinib, dacomitinib) in our centre for advanced EGFR mutated NSCLC between January 2016 and January 2020. We identified 42 patients. Eleven were excluded due to the unavailability of variant allelic frequency data. We reviewed patient records, radiologic and pathology reports to extract clinical and pathological and radiological outcomes. All biopsies were performed at baseline, before the introduction of any therapy. We recorded date of death, if applicable, or the date of the last follow-up visit. All living patients enrolled signed a standardized general research consent form, providing access to their medical records. The study was approved by the regional Ethics Committee (CCER 2020-01628).

We assessed two clinical outcomes, the OS (primary) and PFS (secondary). PFS was calculated from the date of TKI initiation to that of radiological progression or death. OS was calculated from TKI initiation to the date of death, based on the vital status in February, 2020. Patient characteristics included sex, age at diagnosis, smoking status, performance status (PS) and presence of brain metastases at diagnosis. The patient population has been described previously (15).

### Next-Generation Sequencing

We extracted the data from the clinical sequencing reports that were found in the patient files.

The sequencing of tumor DNA was performed for clinical purposes using the IonAmpliseq Hotspot Panel V2 (ThermoFisher scientific) on an IonTorrent Proton sequencer. The tumor cellularity was estimated on hematoxylin and eosin slides and the mutant allele frequency of EGFR were recorded. Co-occurring mutations present on the Ion Ampliseq Hotspot Panel V2 were also recorded. The pathogenicity of mutations, namely their influence on protein function, was assessed based on international databases: ClinVar, Catalog of Somatic Mutations in Cancer (COSMIC) and oncoKB, as well as their described impact on treatment resistance in current medical literature (15).

Copy number variation analysis using the Oncoscan CNV assay (ThermoFisher) was available for some samples, and allowed us to estimate *EGFR* copy number as normal (2 copies) or gain (more than 2 copies).

### Statistical Analysis

The data were analysed in the R language and environment for statistics (version 4.0.2, https://www.r-project.org). We used Kaplan-Meier survival estimates for visual representation, plotted with the Survminer package (version 0.4.8). Cox proportional-hazards univariable and multivariable models were used to test the association of key variables with progression-free and overall survival by calculating hazard ratios and their corresponding 95% confidence intervals. The Wald test was used to assess the statistical significance of Cox models at the usual α < 0.05. Pearson’s test was used to correlate the visually estimated tumor cellularity with the allelic frequency. Fisher’s exact test was used to compare differences in the distribution of allelic frequency (low or high) between clinically relevant groups.

## Results

The median allelic frequency of the *EGFR* mutation was 0.47 (interquartile range: 0.24-0.65), in accordance with the assumption that it is often clonal. Nevertheless, there was considerable variation between patients, with two tumors having an allelic frequency of less than 0.1 and seven tumors an allelic frequency less than 0.2. The visually estimated median tumor cellularity was 0.63 (interquartile range: 0.5-0.9), which is sufficient for molecular analyses. Interestingly, tumor cellularity was weakly correlated with mutation allelic frequency (Pearson’s rho=0.23, P=0.21).

It should be noted that the *EGFR* allelic frequency was not normally distributed (**Supplementary Figure 1** – density plot) and would therefore not be optimal for use as a continuous variable in Cox survival models. Based on the observed tumor cellularity, we would expect clonal *EGFR* mutations to present an allelic frequency of at least 0.31 on average (average cellularity divided by half), even in the absence of copy number gains, which are common in NSCLC and increase the observed allelic frequency. We therefore used a simple cut-off of 0.30 to separate low from high allelic frequency, as a surrogate marker of early, clonal mutations versus late, subclonal mutations. This cut-off also corresponds to the visual plateau between the two main modes of the allelic frequency distribution (**Supplementary Figure 1** – density plot). As expected in this context, most mutations (22 of 31, 71%) were classified as having a high allelic frequency.

Although this was not a prospective randomized trial, the clinical characteristics of the patients were balanced between the high and low groups. The general characteristics of the cohort from which these patients were drawn has also been previously described (15). Specifically, there was no statistically significant difference between *EGFR* mutation allelic frequency and age (Fisher’s test P=0.456), sex (Fisher’s test P=1.0), PS (Fisher’s test P=0.689), smoking status (Fisher’s test P=0.286) or first line treatment with osimertinib (Fisher’s test P=0.704).

**Figure 1:**
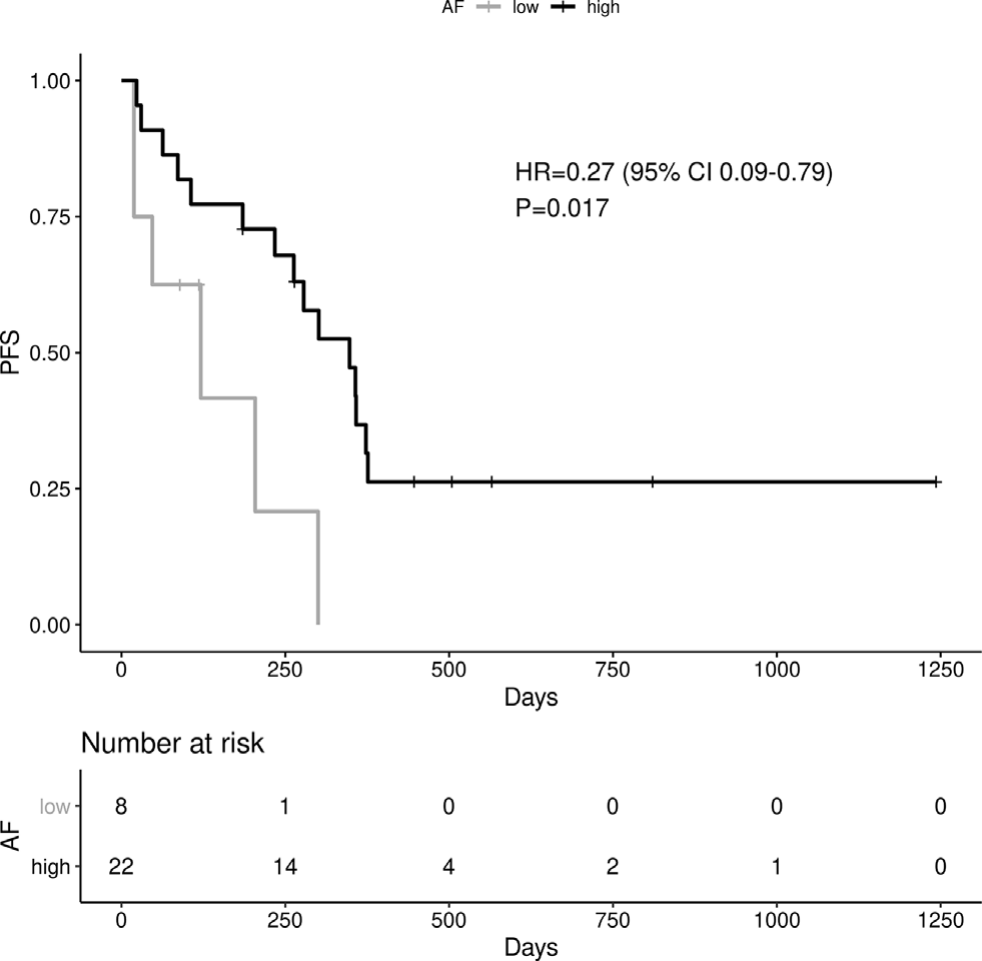
Impact of EGFR allele frequency on PFS.

A high *EGFR* mutation allelic frequency was associated with longer PFS (HR 0.27, 95% 0.09-0.79, P=0.017) (**Figure 1**). This association was robust and persisted even after adjustment for age over 65, sex, smoking and use of osimertinib up-front (13 patients), with a hazard ratio estimate that remained statistically significant in bivariable models (**Table 1A**). It should be noted that only age over 65 was associated with longer PFS in bivariable models with allelic frequency. The statistical significance of the *EGFR* allelic frequency increased further in a multivariable model including the above clinical variables (*EGFR* HR=0.112, 95% 0.023-0.547, P=0.007, **Table 1B**).

**Table 1A:** Bivariable PFS.

Clinical variable	Clinical variable coefficients			EGFR coefficients		
	HR	95% CI	P-value	HR	95% CI	P-value
Age over 65	0.291	0.102-0.830	0.021	0.134	0.037-0.488	0.002
Male sex	2.206	0.898-5.420	0.085	0.337	0.115-0.991	0.048
PS 1 (vs PS0)	0.594	0.195-1.802	0.357	0.300	0.102-0.885	0.029
Current smoker	1.119	0.354-3.533	0.848	0.263	0.087-0.798	0.018
Osimertinib (vs other)	0.402	0.146-1.107	0.078	0.257	0.090-0.738	0.012

**Table 1B:** Multivariable PFS.

Clinical variable	Clinical variable coefficients					
	HR	95 % CI	P-value			
Age over 65	0.268	0.071-1.009	0.052			
Male sex	1.732	0.657-4.566	0.267			
PS 1 (vs PS0)	0.975	0.243-3.917	0.972			
Current smoker	2.750	0.568-13.318	0.209			
Osimertinib (vs other)	0.502	0.149-1.698	0.268			
EGFR high AF	0.112	0.023-0.547	0.007			

**Table 1C:** Bivariable OS.

Clinical variable	Clinical variable coefficients			EGFR coefficients		
	HR	95% CI	P-value	HR	95% CI	P-value
Age over 65	0.859	0.350-2.106	0.740	0.485	0.740-0.485	0.157
Male sex	2.695	1.069-6.795	0.036	0.482	0.036-0.482	0.154
PS 1 (vs PS0)	0.317	0.090-1.124	0.075	0.382	0.075-0.382	0.078
Current smoker	1.199	0.364-3.945	0.765	0.450	0.765-0.450	0.141
Osimertinib (vs other)	0.764	0.240-2.429	0.648	0.483	0.648-0.483	0.153

**Table 1D:** Multivariable OS.

Clinical variable	Clinical variable coefficients					
	HR	95% CI	P-value			
Age over 65	0.664	0.212-2.084	0.483			
Male sex	2.438	0.919-6.467	0.073			
PS 1 (vs PS0)	0.295	0.074-1.185	0.085			
Current smoker	2.426	0.519-11.348	0.260			
Osimertinib (vs other)	0.993	0.275-3.584	0.992			
EGFR high AF	0.319	0.092-1.110	0.073			

A. A summary of bivariable Cox models of PFS including clinical variables (one for each row) and the EGFR allelic frequency as a binary variable (high versus low). B. A multivariable model of PFS including clinical variables and the EGFR allelic frequency as a binary variable (high versus low). C. A summary of bivariable COX models of OS including clinical variables (one in each row) and the EGFR allelic frequency as a binary variable (high versus low). D. A multivariable model of OS including clinical variables and the EGFR allelic frequency as a binary variable (high versus low).

Of the other clinical variables, only male sex was associated with shorter PFS in univariable models. Based on our previous publication, we knew that patients with resistance co-mutation had shorter PFS. For the remaining patients (N=25), without a resistance co-mutation, a high allelic frequency still predicted longer PFS (HR 0.20, 95% 0.04-0.91, P=0.038).

**Figure 2:**
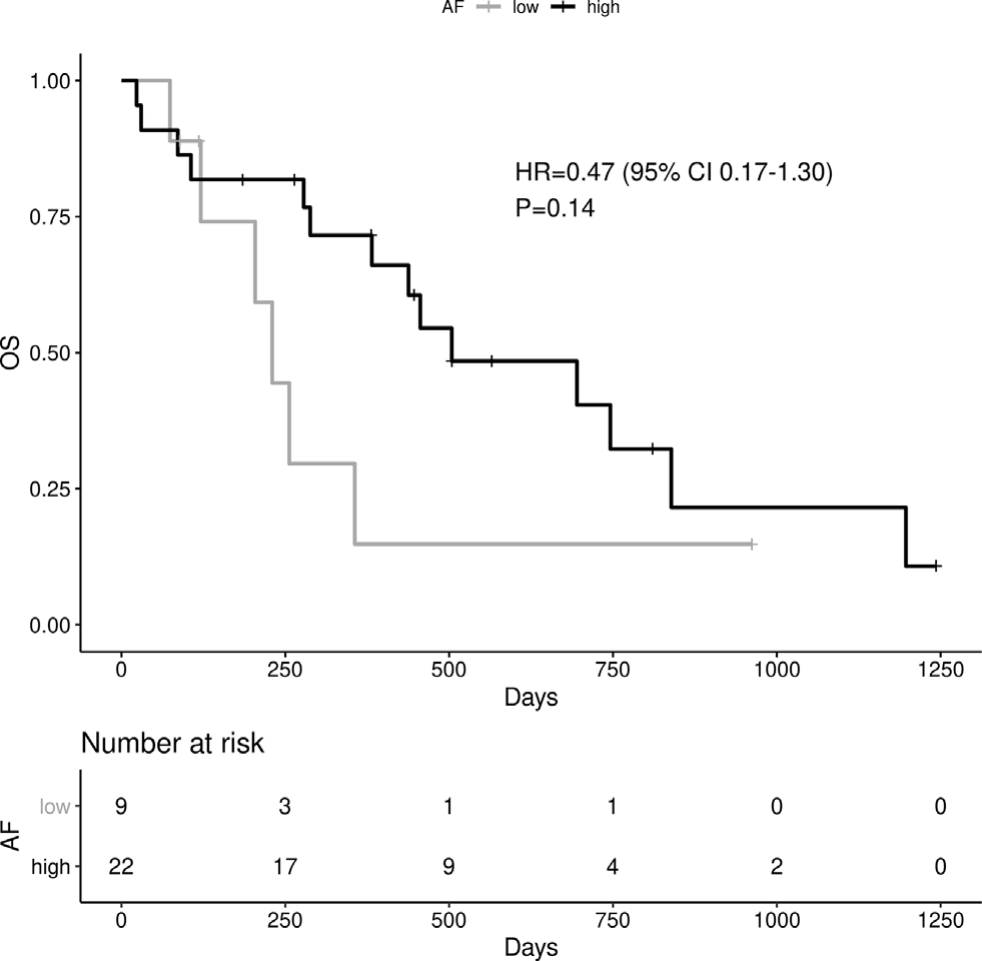
Impact of EGFR allele frequency on OS.

High allelic frequency was not associated with significant difference in overall survival (HR 0.47, 95% 0.17-1.30, P=0.14), despite a visually obvious separation of the Kaplan-Meier curves (**Figure 2**). This result did not change significantly after adjustment for clinical variables of interest in bivariable models (**Table 1C**). Even though the *EGFR* hazard ratio improved in a multivariable model with clinical variables, it did not attain significance, remaining a statistical trend (*EGFR* HR=0.319, 95% 0.09-1.11, P=0.073 **Table 1D**).

As noted above, the allelic frequency was not normally distributed. Even after log transformation, the martingale residuals showed a nonlinear fit against PFS in a Cox model (**Supplementary Figure 2** – martingale pfs). Nevertheless, when the log-transformed *EGFR* allelic frequency was used as a continuous variable, the association with PFS remained consistent (HR=0.589, 95% 0.35-0.99, P=0.0452). Again, there was no association with OS (HR=0.638, 95% 0.37-1.1, P=0.114). Both of these results should be considered exploratory.

The *EGFR* copy number can influence the allelic frequency of the mutation. Indeed, in our data, most tumors with high allelic frequency also had copy number gains (15/20), while tumors with low allelic frequency typically did not have such gains (2/9). This difference was significant (Fisher’s test OR=9.51, P=0.014). Despite this observation, the presence of copy number gains in *EGFR* did not predict PFS (HR=0.578, 95% 0.24-1.41, P=0.229) or OS (HR=0.537, 95% 0.22-1.34, P=0.182).

By combining the presence of resistance co-mutations in other cancer-related genes with the allelic frequency of the *EGFR* mutation, we hypothesized that we would more accurately capture the driver status of *EGFR* and predict treatment response. Even though resistance co-mutations in other genes were more often associated with low *EGFR* mutation allelic frequency (3 of 5), the very small numbers preclude a statistically significant conclusion (Fisher’s test OR 4.7, P=0.131). In that sense, the information obtained from co-mutations and allelic frequency appears to be complementary. We therefore defined a “sensitive” tumor as one that did not harbor resistance co-mutations and in which the *EGFR* mutation had a high allelic frequency. Under that definition, 20 of 31 tumors were classified as sensitive (64%), compared with 88% when only the co-mutation was considered.

**Figure 3:**
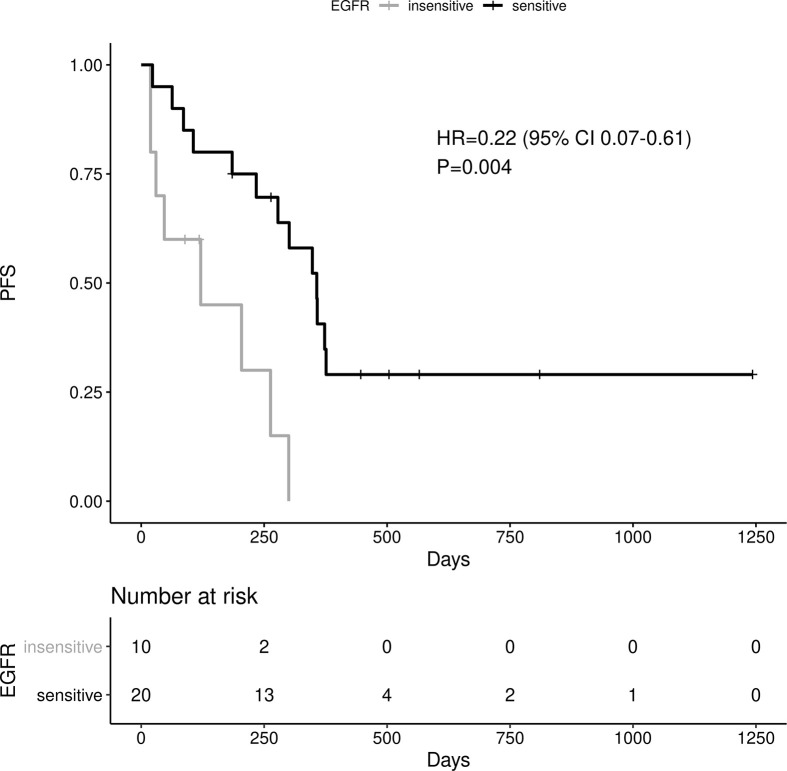
Impact of tumor sensitivity on PFS.

**Figure 4:**
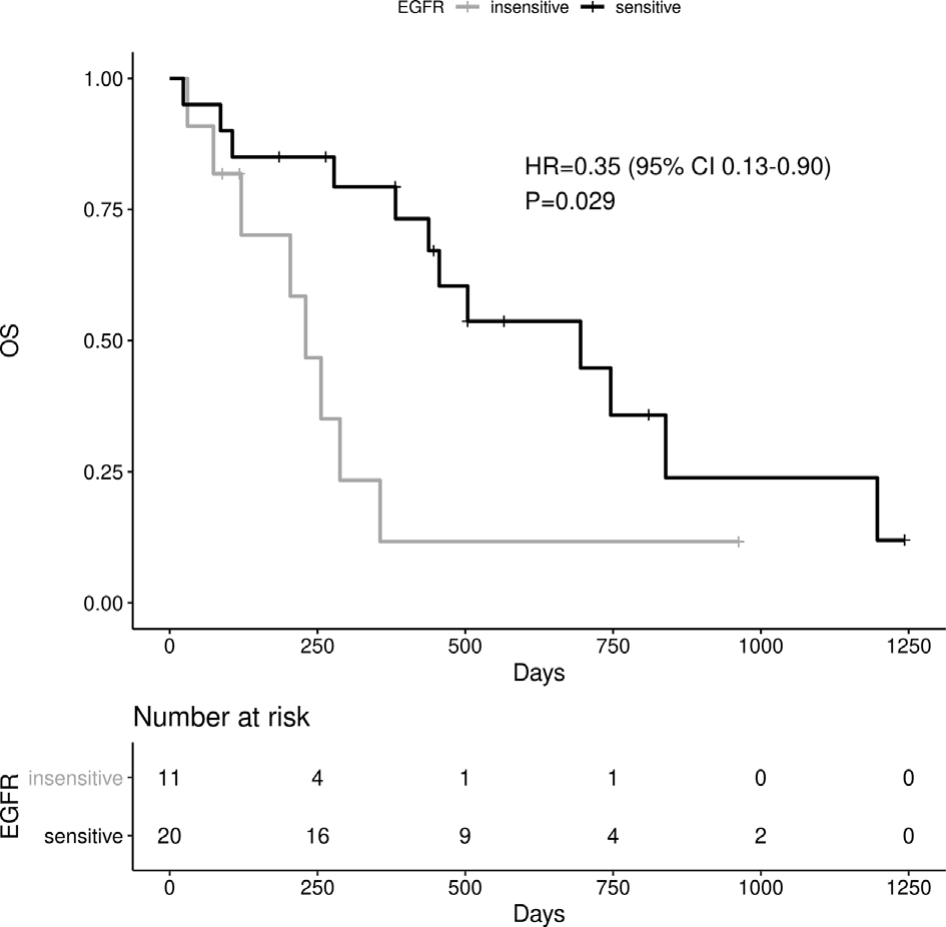
Impact of tumor sensitivity on OS.

Sensitive tumors were associated with significantly longer PFS (HR 0.22, 95% 0.07-0.61, P=0.004) and OS (HR 0.35, 95% 0.13-0.90, P=0.029) (**Figures 3** and **4**). The association with both PFS and OS remained significant after adjustment for clinical variables in a series of bivariable models with age over 65, sex, performance status, smoking and osimertinib use upfront (**Table 2**). Furthermore, EGFR sensitivity remained significantly associated with PFS (HR=0.137, 95% 0.04-0.51, P=0.003) and OS (HR=0.196, 95% 0.06-0.69, P=0.011) in multivariable models including all the above variables. Of the other clinical variables, only male sex was predictive of shorter OS in the multivariable model (HR=2.707, 95% 1.00-7.31, P=0.049). Twelve-month PFS was 0% in the insensitive group, compared with 41% in the sensitive group. At one year, OS was 10% in the insensitive group, compared with 79% in the sensitive group. Patients with insensitive tumors were almost 10 times more likely to die before 12 months than patients with sensitive tumors (Fisher’s test OR 9.7, P=0.007).

**Table 2A:** Bivariable PFS.

Clinical Variable	Clinical variable coefficients			EGFR sensitive		
	HR	95% CI	P-value	EGFR HR	95% CI	P-value
Age over 65	0.415	0.171-1.005	0.051	0.174	0.059-0.509	0.001
Male sex	2.208	0.906-5.385	0.081	0.251	0.088-0.718	0.010
PS 1 (vs PS0)	0.671	0.217-2.076	0.488	0.237	0.081-0.698	0.009
Current smoker	1.298	0.398-4.237	0.666	0.200	0.066-0.606	0.004
Osimertinib (vs other)	0.453	0.164-1.251	0.127	0.229	0.081-0.646	0.005

**Table 2B:** Multivariable PFS.

Clinical variable	HR	95% CI	P-value			
Age over 65	0.369	0.123-1.109	0.076			
Male sex	2.404	0.923-6.264	0.073			
PS 1 (vs PS0)	1.036	0.248-4.335	0.961			
Current smoker	3.099	0.610-15.750	0.173			
Osimertinib (vs other)	0.562	0.160-1.975	0.369			
EGFR sensitive	0.137	0.037-0.507	0.003			

**Table 2C:** Bivariable OS.

Clinical Variable	Clinical variable coefficients			EGFR sensitive		
	HR	95% CI	P-value	EGFR HR	95% CI	P-value
Age over 65	0.955	0.382-2.386	0.922	0.350	0.132-0.930	0.035
Male sex	2.806	1.105-7.123	0.030	0.335	0.128-0.879	0.026
PS 1 (vs PS0)	0.311	0.087-1.112	0.072	0.283	0.099-0.808	0.018
Current smoker	1.483	0.427-5.145	0.535	0.305	0.106-0.877	0.028
Osimertinib (vs other)	0.843	0.262-2.711	0.775	0.354	0.135-0.926	0.034

**Table 2D:** Multivariable OS.

Clinical variable	HR	95% CI	P-value			
Age over 65	0.596	0.187-1.903	0.382			
Male sex	2.707	1.003-7.305	0.049			
PS 1 (vs PS0)	0.268	0.066-1.084	0.065			
Current smoker	3.536	0.667-18.731	0.138			
Osimertinib (vs other)	1.189	0.316-4.471	0.798			
EGFR sensitive	0.196	0.055-0.693	0.011			

(A) A summary of bivariable Cox models of PFS including clinical variables (one for each row) and the EGFR sensitivity as a binary variable (sensitive vs insensitive). (B) A multivariable Cox model of PFS including clinical variables (one for each row) and the EGFR sensitivity as a binary variable (sensitive vs insensitive). (C) A summary of bivariable COX models of OS including clinical variables (one in each row) and the EGFR sensitivity as a binary variable (sensitive vs insensitive). (D) A multivariable Cox model of OS including clinical variables (one for each row) and the EGFR sensitivity as a binary variable (sensitive vs insensitive).

## Discussion

Biologically, it could be surmised that increased allelic frequency would be correlated with an increased probability of the variant being an oncogenic driver. There is not much literature assessing the impact of EGFR allelic frequency in NSCLC, and further complicating matters, available data are discordant. In addition, several studies are based on circulating tumor DNA and comparisons with tissue biopsies may not be always appropriate.

A retrospective analysis from the Shizuoka Lung Cancer Mutation Study found that among 705 enrolled patients, 102 lung adenocarcinoma patients carried the typical EGFR L858R exon 21 mutation (18). Forty-eight patients were assessed both by pyrosequencing, a non-electrophoretic real-time sequencing approach, and by outsourced polymerase chain reaction (PCR) laboratory tests. Among these patients, the median mutant allelic frequency was 18.5% (8% to 82%). Receiver operating characteristic curves found that a mutant allelic frequency of 9% resulted in 100% sensitivity and 99% specificity. The authors then used this cut-off to ass the impact of allelic frequency on survival in patients receiving TKI. The PFS among patients with a mutant allelic frequency ≤9% was 92 days, compared to 284 days for those with a frequency greater than 9% (p=0.0027), suggesting a predictive role of this variable. It should be noted that this study did not analyze allelic frequency among patients with EGFR exon 19 deletions, as this mutation was only detected qualitatively using fragment analysis in this initiative.

A further retrospective trial using digital droplet PCR based performed analyses on archived tissue from 233 lung cancer patients treated with first-generation EGFR TKIs (19). The results supported previous finding, as the authors found a correlation between mean allele frequency and clinical outcomes. Among patients with a partial response, the mean allele frequency was 48.6%, while it was 27.4% in patients with stable disease and 9.5% among those with progressive disease (p for partial response versus disease stability: 0.0078, partial response versus progressive disease: 0.000001, stable disease versus progressive disease 0.029).

The largest available dataset based on a prospective study is an unplanned retrospective analysis from the phase III CTONG 0901 trial, which compared the efficacy of erlotinib to gefitinib in advanced NSCLC harboring EGFR exon 19 or 21 mutations, measured by NGS. Among 194 patients with EGFR mutant NSCLC, the median mutant allelic frequency was 25.8%, with a range of 1.4% to 86.2%. Patients were divided into two groups, high mutant allelic frequency (25.8 to 86.2%) and low allelic frequency (1.4 to 25.8%). The authors evaluated ORR, PFS and OS. The first ORR did not differ between groups, at 56.2% and 57.5% in the high and low groups, respectively. Similarly, there was no difference in PFS, at 11.2 and 12.4 months (*P* = 0.509) nor OS at 20.5 and 23.1 months (*P* = 0.500), in the high and low allelic frequency groups, respectively (20).

Among patients receiving osimertinib in second-line with a T790M resistance mutation after failure of an earlier generation EGFR TKI, the maximum EGFR somatic allele frequency of EGFR variants measured in circulating tumor DNA does not appear to predict response rate or survival. However, the ratio of T790M allele frequency to maximum EGFR somatic allele frequency is highly predictive of ORR (p=0.002) and PFS (p=0.006) (21). A retrospective analysis on 54 patients mirrored these results (21). Both of these retrospective analyses are supported by a recent prospective trial on 34 patients who progressed on first or second-generation TKIs and developed T790M resistance mutations (22). After enrolment, there was a baseline plasma sample and patients started osimertinib. Cell-free DNA was analyzed by digital droplet PCR to calculate the mutant allele frequency. Patients with higher non-T790M mutant EGFR allele frequencies fared less well than those with lower frequencies, while higher T790M ratios provided superior PFS (6 months versus not reached, p=0.01). These studies highlight the potential predictive value of mutant allelic frequency in the second-line setting.

In our cohort of patients with EGFR-mutant NSCLC treated with targeted therapy in the front-line setting, the variant allelic frequency is associated with survival outcomes, whether they are receiving first or third generation TKIs. While our cohort is small in size, there is a clear PFS improvement and trend toward OS benefit among patients whose disease harbors a mutant allelic frequency greater than 0.3, or 30%. This appears to be an independent predictive factor for outcomes in our bivariable model.

Furthermore, after correcting for the presence of co-occurring pathogenic mutations, known to predict inferior outcomes in this population, the difference remains significant. Combining both predictive factors differentiates patients into very distinct prognostic groups. One could question the role of allelic frequency given the stronger predictive impact of resistance co-mutations; however, it is important to note that co-mutations are rare. By combining the two factors, we classify 64% of tumors as likely to be sensitive to EGFR TKIs, revealing 36% prone to respond less favorably to therapy. When we consider co-mutations alone, only 12% are classified as insensitive. The latter have a greater association with overall prognosis, but the former may have a more meaningful clinical impact, due to its wider applicability and role in predicting the efficacy of front-line TKIs. The combination of these two was consistently associated with both PFS and OS in univariable, bivariable and multivariable analyses and the magnitude of the effect was clinically very significant.

*EGFR* variant allelic frequency was driven by copy number status but did not correlate with the visually estimated tumor cellularity in our data. We believe that the allelic frequency also captures the proportion of cells carrying the mutation and is therefore able to separate tumors with a truncal mutation, which are more likely to respond favorably, from tumors with subclonal *EGFR* alterations, which are more likely resistant. In that sense, we feel that the *EGFR* variant allelic frequency can be a useful biomarker in the clinic. 

The small sample size is a limitation of the interpretation of our results. Despite the sample size and the lack of randomization, the data appear balanced between groups, especially with respect to the use of osimertinib as a first line, which could be a potential confounder.

There is no well-established allelic frequency cut-off to classify EGFR mutations and no clear method for deriving the cut off. Our choice of cut-off is based on the visual estimation of tumor cellularity by an experienced molecular pathologist and the expectation that a homogeneous population of EGFR mutant cells would produce an allelic frequency of at least half the tumor cellularity, corresponding to one mutant allele out of two alleles (the other being normal). There are situations where this may not occur for other reasons, such as the amplification of the normal allele, but there is no selection pressure in favor of the normal allele. It is unclear whether this cut-off will translate to other cohorts, but our assumptions are general and not specific to our institution or the period of data collection.

Finally, we do not have TMB estimates for most of our patients. This could be relevant as high TMB is known to be associated with poor prognosis in patients whose cancer harbors an EGFR mutation (23).

## Conclusion

The mutant allelic frequency of EGFR in NSCLC appears to be associated with clinical outcomes among patients treated with TKIs. In spite of our small cohort size, we note a clear PFS improvement in patients with a high EGFR allelic frequency compared to those with a low frequency. There is a clear trend toward improved OS, though it is not significant. This predictive biomarker is independent of the generation of TKIs used and of the presence of resistance co-mutations. Interestingly, by combining the latter with variant allelic frequency, we identify two clear prognostic groups, resistant and sensitive patients to TKIs. The complementary nature of these analyses and clinical implications of our results require further validation.

## Data Availability Statement

The data were obtained from the patient record of the university hospital of Geneva and are not public. We are willing to share the extracted, de-identified data upon reasonable request. The primary sources (documents from the digital patient record) cannot be released, due to the presence of identifying information.

## Ethics Statement

The studies involving human participants were reviewed and approved by EC Geneva. The patients/participants provided their written informed consent to participate in this study.

## Author Contributions

All the authors contributed equally to the manuscript. All authors contributed to the article and approved the submitted version.

## Conflict of Interest

The authors declare that the research was conducted in the absence of any commercial or financial relationships that could be construed as a potential conflict of interest.

## Supplementary Material

Supplementary Figure 1
*EGFR* allelic frequency distribution density plot.

Supplementary Figure 2 Martingale PFS.
