# Supplementary figures and images for: The Impact of Variant Allele Frequency in EGFR Mutated NSCLC Patients on Targeted Therapy
Source: Front Oncol. 2021 Mar 30;11:644472. doi: 10.3389/fonc.2021.644472 (PMC8044828; doi:10.3389/fonc.2021.644472)

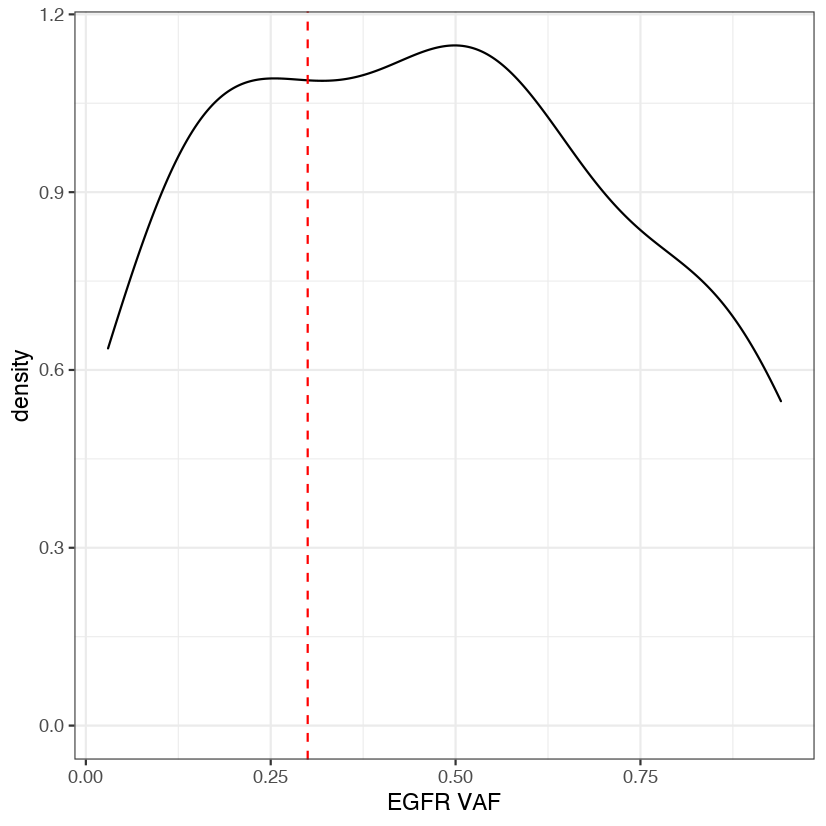

Supplement: Supplementary Figure 1 — EGFR allelic frequency distribution density plot. [file Image_1.tif]

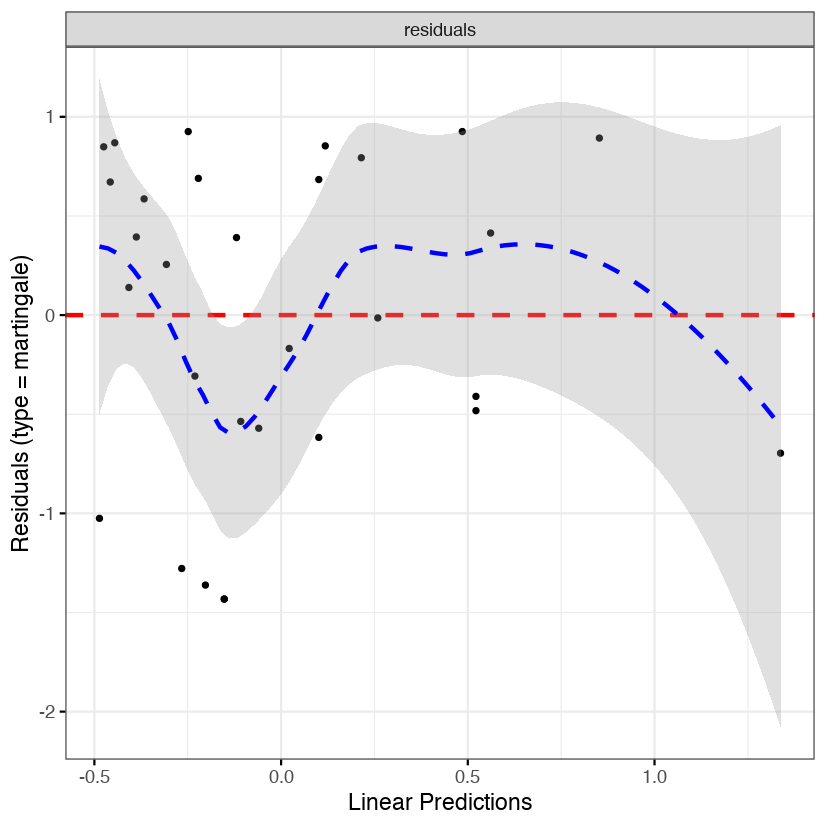

Supplement: Supplementary Figure 2 — Martingale PFS. [file Image_2.tif]
